# Extra Virgin Olive Oil Protects the Testis and Blood from the Toxicity of Paracetamol (Overdose) in Adult Male Rats

**DOI:** 10.3390/biology10101042

**Published:** 2021-10-14

**Authors:** Latifa Ishaq Khayyat

**Affiliations:** Biology Department, Faculty of Applied Sciences, Umm Al-Qura University, Makkah, Saudi Arabia; likhayyat@uqu.edu.sa; Tel.: +966-66561544832; Fax: +966-125272089

**Keywords:** extra virgin olive oil, paracetamol, hematotoxicity, testicular toxicity, fertility, antioxidant

## Abstract

**Simple Summary:**

In rats, as in humans, paracetamol drug is used for analgesic and antipyretic therapy. Overdoses or long-term use of paracetamol cause toxicity effects in the body organs. Extra virgin olive oil has beneficial effects on health, offering protection against immunotoxicity, genotoxicity, and cytotoxicity. This study investigates the protective effect of Extra virgin olive oil against blood toxicity and testis toxicity induced by paracetamol overdose in rats. The histological, ultrastructural, and biochemical results obtained in the present study showed the protected effect of the antioxidant and phenolic components in Extra virgin olive oil against toxicity induced by Paracetamol overdose.

**Abstract:**

Extra virgin olive oil (EVOO) is important in people’s daily diets. Paracetamol is a widely used analgesic and antipyretic drug. The aim of this study is to investigate the protective effect of EVOO against hematotoxicity and testicular toxicity induced by paracetamol overdose in rats. Forty rats were divided into four groups. Group 1 rats were given water (control), Group 2 rats were given oral EVOO daily (2 mL/kg b.wt.), Group 3 rats were given oral paracetamol daily (650 mg/kg b.wt.), and Group 4 rats were given paracetamol and EVOO daily. After 15 days, blood and testis samples were collected for biochemical, histological, and ultrastructural studies. The results show that paracetamol decreased the PCV, Hb, and RBC counts relative to the control, and significantly increased the WBC counts and stab cells in Group 3. A significant decrease in blood testosterone was found in Group 3 compared to the control, while a significant increase in testosterone levels was observed in Group 4 compared to Group 3. Light and electron microscopy showed disorganized seminiferous tubules in Group 3. The testis in Group 4 appeared in normal shape. In conclusion, the results indicate that EVOO protects the testis and blood from paracetamol toxicity and may also increase fertility in male rats.

## 1. Introduction

Paracetamol and acetaminophen are two official names for the same chemical compound. They are derived from its chemical name: N-acetyl-para-aminophenol. Paracetamol was introduced to the pharmacological market as a prescribed antipyretic and analgesic drug under the trade names Panadol and, for children, Tylenol [1]. Paracetamol is a nonprescription drug, and it is safe at therapeutic doses for analgesic and antipyretic therapy. Until now, its mechanism of action has been unclear. Paracetamol is the drug of choice for patients who cannot use nonsteroidal anti-inflammatory drugs (NSAIDs) because of hypersensitivity to aspirin, gastric ulcers, impaired blood coagulation, pregnancy, breastfeeding, or fever accompanying disease in children [1].

Overdoses or the long-term use of paracetamol causes toxicity effects in the organs, including hepatotoxicity, renal toxicity, and testicular toxicity, and affects the blood chemistry and reproductive parameters [2,3,4,5,6].

High doses of paracetamol appear to affect the male reproductive system, changing the semen quality, particularly the sperm morphology and, hence, its fertilizing ability. Paracetamol may affect the semen quality by suppressing testosterone synthesis, inducing oxidative stress, provoking the apoptosis of spermatocytes, reducing nitric oxide production, and inhibiting prostaglandin synthesis [7].

Many studies show that overdoses and long-term paracetamol use increases the risk of oxidative stress, damaging hepatocytes, renal tubules, testicular tubules, and blood cells, and causing hypertension and heart infarction [2,3,4,5,6,7].

Several studies worldwide have examined the role of medicinal plants in complementary medicine. Extra virgin olive oil (EVOO) is important in people’s daily diets. Olive oil is beneficial because of its high oleic acid content and the antioxidant potential of its polyphenols. Extra virgin olive oil has beneficial effects on health, offering protection against immunotoxicity, genotoxicity, and cytotoxicity, and decreasing the risk of developing chronic diseases, such as cancer and heart disease [8,9,10,11,12]. Its health benefits are related to both its fatty acid composition and minor compounds, including tocopherols, polyphenols, sterols, and carotenoids. Other studies show that polyphenolic compounds exhibit strong radical scavenging activity and appear to be more effective than other important dietary antioxidants [13,14].

Compared to other oils, virgin olive oil contains a higher proportion of monounsaturated fatty acids, which decreases the levels of free radicals, thereby reducing the damage caused by oxidative stress. Oxidative stress is responsible for many damaging effects that can cause several diseases, such as cancer, atherosclerosis, and neurodegenerative diseases [15,16].

Extra virgin olive oil showed a protective effect against paracetamol hepatotoxicity induced by paracetamol overdose and nephrotoxicity in male rats [5,6]. Olive oil also ameliorates testicular damage and protects the reproductive organs from cadmium toxicity by reducing oxidative stress [17]. The effects of virgin olive oil on blood pressure and the renal aminopeptidase activities in male Wistar rats were studied by comparing high saturated fat diets that have been associated with the development of obesity and hypertension. Virgin olive oil is characterized by its high content of monounsaturated fatty acids and is used as a dietary factor capable of positively regulating cardiovascular function. Moreover, the effects of virgin olive oil have been linked to changes in the local renal renin-angiotensin system (RAS) and the activity of the sympathetic nervous system. The results show that extra virgin olive oil has a protective effect on systolic blood pressure and seems to have an indirect effect on the sympathetic system and the metabolic activity in the kidney [18].

A study with diabetic rats showed that extra virgin olive oil can significantly increase the high-density lipoprotein content, and significantly decrease the serum total cholesterol, triglyceride, and low-density lipoprotein content in serum [19]. Virgin olive oil (VOO) is thought to play a protective role against cardiovascular disease. The effects of VOO and phenol-enriched VOO on lipoprotein atherogenicity and HDL atheroprotective properties were described by Farràs et al. [20]. Phenol-enriched VOO is a strategy for increasing VOO phenolic content without increasing its fat content, thus enhancing the potential of VOO to improve lipoprotein functions. In long-term studies, the VOO reduces oxLDL levels while enhancing its resistance to oxidation. The improvement in lipoperoxidation is closely associated with the OO phenolic content. Moreover, VOO enriched with OOPC reduces the number of LDL-P and atherogenic small LDL particles. On the other hand, the OO showed an improvement in the postprandial lipemia, but only in patients with type 2 diabetes. In addition, VOO and phenol-enriched VOO, showed several enhanced HDL-mediated atheroprotective functions, including the ability to stimulate macrophage cholesterol efflux and HDL antioxidants, as well as anti-inflammatory properties. Some of these changes are related to the dietary VOO effects on the HDL size, the increase in HDL antioxidant enzymes, and the upregulation of several transporters involved in cholesterol efflux. Many studies clarify the mechanism behind the protective effects of EVOO phenols on the cardiovascular system, which suggests the role of hydroxytyrosol and secoiridoid molecules [21,22,23].

**Figure 1:**
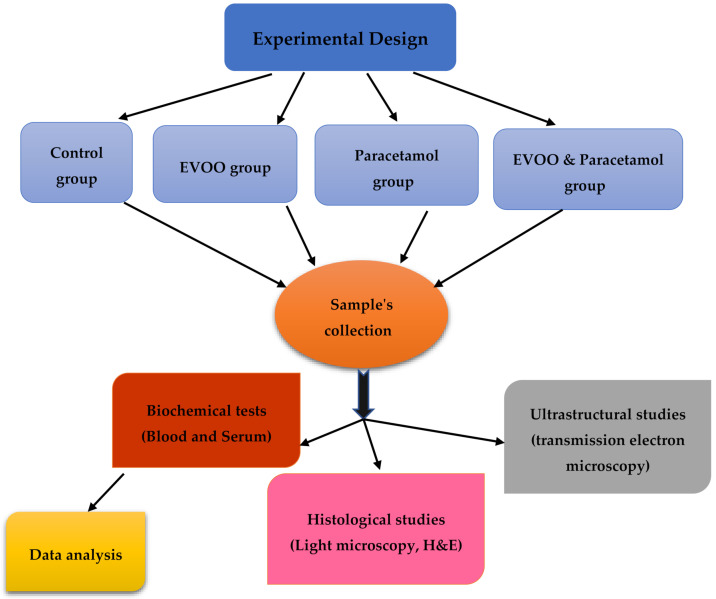
A flowchart showing the overall study approach.

The present study aims to investigate the protective effect of EVOO against toxicity in the blood and testis induced by paracetamol overdoses in adult male rats. Figure 1 shows a flowchart showing the overall study approach.

## 2. Materials and Methods

### 2.1. Animals

Forty adults male Wistar albino rats (146–156 g) were used in the present study. They were maintained in special standard metallic cages (five rats per cage) and kept under standard laboratory conditions in a temperature-controlled environment (24 ± 2 °C), with an alternating 12-h light–dark cycle, and relative humidity of 50 ± 5%, and acclimatized for one week prior to the study. They were given free access to a commercial balanced stock diet and water. The experiments were conducted in compliance with the Guide for the Care and Use of Laboratory Animals. Our experimental procedures were approved by the Menoufia University IACUC Committee for Care of Laboratory Animals (Approval No.: MNSP155).

Paracetamol was obtained from Al Nahdi, a local pharmacy in Makkah, Saudi Arabia (Panadol 665, GlaxoSmithKline Australia Pty Limited, Boronia, Australia). The EVOO was purchased from Bin Dawood local market in Makkah, Saudi Arabia.

### 2.2. Experimental Design

The animals were randomly divided into four groups (*n* = 10).

Control Group: The animals were given distilled water orally at a daily rate of 2 mL/kg b.wt. for 15 days.

EVOO Group: The animals were given EVOO orally at the daily rate (2 mL/kg b.wt.) through a gastric tube for 15 days (positive control group) [24].

Paracetamol Group: The animals were given paracetamol orally at the daily rate (650 mg/kg b.wt.) through a gastric tube for 15 days [25]. 

Paracetamol-with-EVOO Group: The animals were given paracetamol orally at the daily rate (650 mg/kg b.wt.), and EVOO orally at the daily rate (2 mL/kg b.wt.) through a gastric tube for 15 days. 

Animals from both the control and treatment groups were killed by cervical dislocation after 15 days of treatment. Blood samples were collected from each rat via cardiac puncture and maintained in special tubes containing ethylenediaminetetraacetic acid (EDTA) for hematological studies.

For the histological and ultrastructural studies, small samples of testis were removed quickly and fixed in a convenient fixative. 

### 2.3. Biochemical Tests

Blood samples were collected from each rat via a cardiac puncture method and allowed to clot. The serum was rapidly separated by centrifuging the clotted blood at 3000× *g* for 10 min in a Beckman Model T-6 refrigerated centrifuge, and then stored in clean and dry tubes. Sera were stored at −20 °C until assayed for the biochemical parameters [24,25,26].

For the blood hematological studies, blood samples were collected from each rat via cardiac puncture and maintained in special tubes containing ethylenediaminetetraacetic acid (EDTA).

### 2.4. Histological and Ultrastructural Studies 

For the histological studies, small samples of testis from all groups were removed quickly and fixed in Bouin’s solution. The fixed tissues were embedded in paraffin wax. Sections of 5 µm were stained with hematoxylin and eosin (H & E dye) for light microscopy examination. 

For the transmission electron microscopy, small pieces of testis were immediately fixed in 4F1G in phosphate buffer (pH 7.2) for 3 h at 4 °C, then post-fixed in 2% OsO_4_ in the same buffer at 4 °C for 1–2 h. The specimens were dehydrated through a graded series of ethanol, embedded in an Epon-Araldite mixture, and polymerized at 60 °C. Ultrathin sections (50 nm) from selected areas were cut with glass knives on an LKB ultramicrotome double-stained with uranyl acetate and lead citrate, and examined with a Jeol 100CX electron microscope. Table 1 shows the morphological parameters in the cell study.

### 2.5. Statistical Analysis

The data were presented as the mean ± SD of ten replicates and were analyzed by a one-way ANOVA and LSD post hoc tests using SPSS software. The results were considered statistically significant when *p* < 0.05.

## 3. Results 

### 3.1. Histological Results 

**Figure 2:**
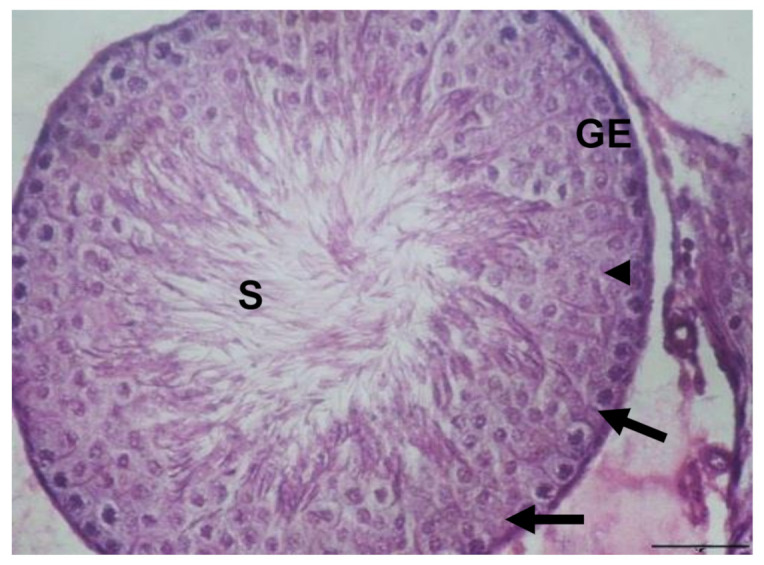
Section of testis in control group rats showing normal structure of seminiferous tubules with normal germinal epithelium (GE), Sertoli cells (arrow), and sperm (S) (×400).

**Figure 3:**
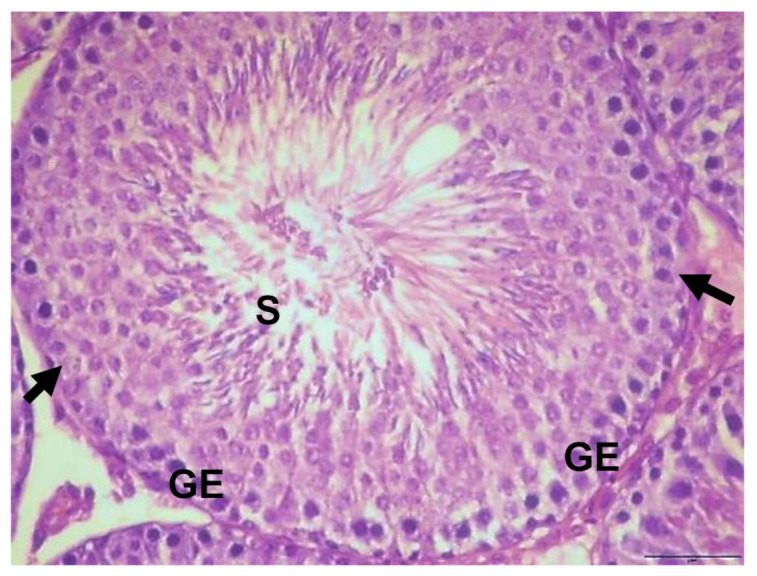
Section of testis of rats treated with EVOO for 15 days showing normal seminiferous tubules with normal germinal epithelium, Sertoli cells (arrow) (GE), and sperm (S) (×400).

Light microscopy examination of the testis sections of the control rats showed the typical features of normal seminiferous tubules, with normal spermatogenic cells, Sertoli cells, and spermatozoa (Figure 2). The testicular tissue of rats given EVOO for 15 days showed no obvious changes compared to the control group. The seminiferous tubules appeared with normal spermatogenic cells, sperm, and Sertoli cells (Figure 3).

**Figure 4:**
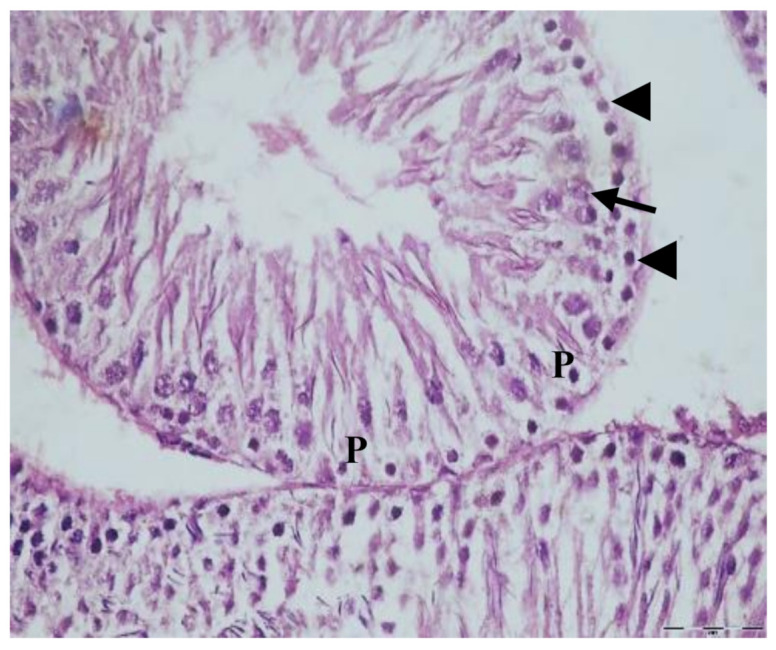
Section of testis of rats treated with paracetamol for 15 days showing disorganized arrangement of spermatogenic cysts (arrows), separated and ruptured basement membrane of the germinal epithelial cells (head arrows), degenerated germ cells with pyknotic nuclei (P) (H&E ×400).

**Figure 5:**
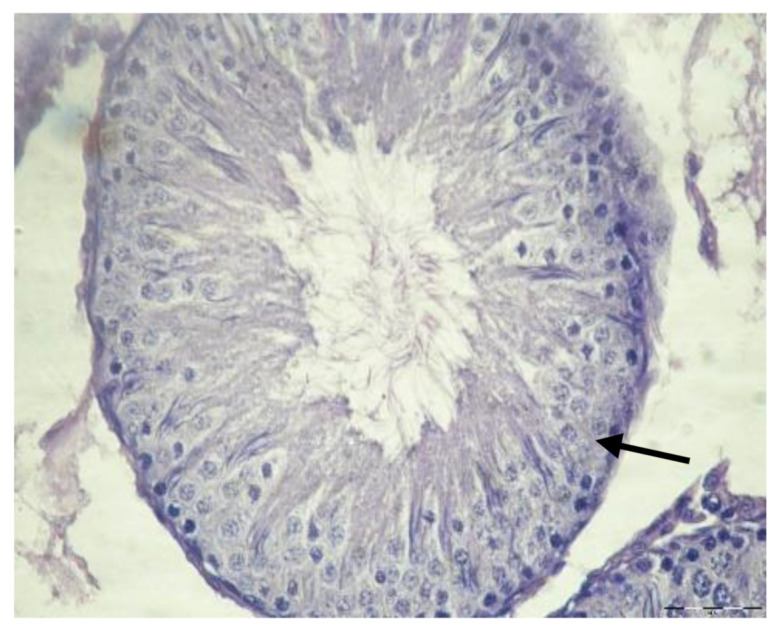
Section of testis of rats treated with EVOO and paracetamol for 15 days showing most seminiferous tubules with normal structure (arrow) (×400).

The testis sections of animals given paracetamol for 15 days showed testicular distortion compared to the controls. Loss of the normal testicular structure, with markedly disorganized spermatogenic cysts with separated and ruptured basement membranes of the germinal epithelial cells, and degenerated germ cells with pyknotic nuclei, are clearly observed (Figure 4). Moreover, the testis sections of the rats treated with EVOO and paracetamol for 15 days showed improvement in most seminiferous tubules and less prominent histopathological alterations compared to the paracetamol group (Figure 5).

### 3.2. Electron Microscopy Results

**Figure 6:**
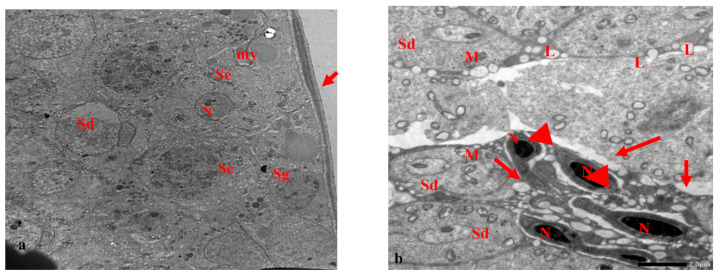
(**a**,**b**): Electron micrograph of a section of rat’s testis in the control group. (**a**) showing normal seminiferous tubule with normal spermatogenic epithelium (arrow), spermatogonia (Sg), primary spermatocytes (Sc), and spermatids (Sd). Sertoli cell (Se) appeared with the nucleus (N) and the basement membrane enclosing myoid cell (my) (×1000). (**b**): showing normal sperms. The sperms have flagellum (head arrow) and an elongated condensed nucleus (N) and an acrosomal cap (arrow). The spermatids appeared (Sd) with the nucleus and cytoplasm rich in mitochondria (M), and lipid droplets (L) between the cells (×2500).

Electron micrographs of the testis of the control rats show the normal structure of seminiferous tubules. These are lined with spermatogenic epithelial cells, followed by the usual sequence of spermatogonia, primary spermatocytes, and spermatids. Spermatogenic cells appear with normal nuclei containing peripheral clumped chromatin. The primary spermatocytes appear above the spermatogonia as large round cells. The spermatids appear with round nuclei and dark clumps of heterochromatin and cytoplasm rich in mitochondria, with lipid droplets between the cells (Figure 6a,b). Sertoli cells appear with normal nuclei and the basement membranes enclosed by myoid cells (Figure 6a). The mature spermatozoa were observed with acrosomes at the head, and condensed nuclei, neck, and midpieces with mitochondrial sheaths (Figure 6b). 

**Figure 7:**
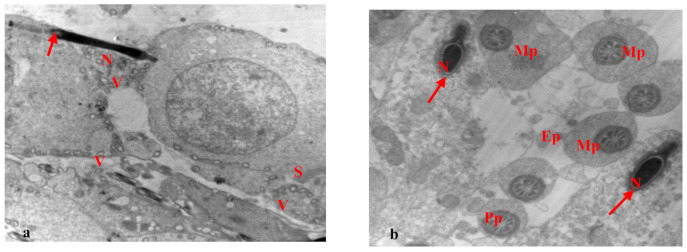
(**a**,**b**): Electron micrograph of a section of rat’s testis, treated with EVOO for 15 days. (**a**) showing normal seminiferous tubule with normal spermatids (Sd) and a normal nucleus. Sperm appeared with an elongated condensed nucleus (N) and an acrosomal cap (arrow) at the leading point of the head. Notice vacuoles (v) in cells (×2000). (**b**) showing that most of the different parts of the sperm flagellum have normal structure, midpiece (Mp), principal piece (Pp), and end piece (Ep), with the typical arrangement of flagellar axoneme. Sperms have normal flagellum and elongated condensed nucleus (N) and an acrosomal cap (arrow) (×1500).

Electron micrographs of testis of rats treated with EVOO for 15 days showed the normal seminiferous tubules with normal spermatids and normal nuclei. Sperm appeared with elongated condensed nuclei and acrosomal caps at the fronts of the heads. Vacuoles were observed in the cells (Figure 7a). The sperm had normal flagella-elongated condensed nuclei and acrosomal caps. Most sections of the sperm flagella had normal structures, comprising midpieces, principal pieces, and end pieces, with the typical arrangements of flagellar axonemes (Figure 7b). 

**Figure 8:**
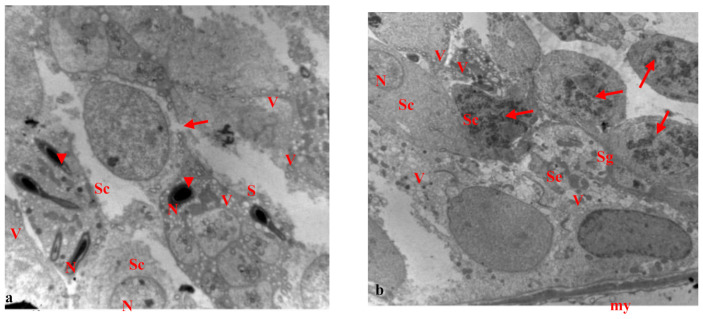
(**a**,**b**): Electron micrograph of a section of rat’s testis treated with paracetamol for 15 days. (**a**) showing seminiferous tubule with several changes, primary spermatocytes (Sc) with pyknotic nucleus (N), and the rupture of the cell membrane (arrow). Some sperm without flagellum (head arrow) and elongated condensed abnormal nucleus (N). Notice the wide separation (s) between neighboring cells and vacuoles (v) in most cells (×1500). (**b**) showing seminiferous tubules with several changes, Sertoli cell (Se) appeared with abnormal chromatin in nucleus (N), and the basement membrane enclosing myoid cell (my). Most of the spermatogonia (Sg), and primary spermatocytes (Sc) with chromatin condensation in their nucleus, primary spermatocytes (Sc) with necrotic nucleus (N). Notice vacuoles (v) in cells (×1000).

However, the testis of rats treated with paracetamol for 15 days showed seminiferous tubules with several changes, primary spermatocytes with pyknotic nuclei, and ruptured cell membranes. Some sperm had missing flagella with elongated and condensed abnormal nuclei. Moreover, wide separations were observed between neighboring cells and vacuoles for most cells (Figure 8a). Several changes appeared in the seminiferous tubules. Sertoli cells appeared, with abnormal chromatin in their nuclei and basement membranes, enclosing myoid cells. Most of the spermatogonia and primary spermatocytes showed condensed chromatin in their nuclei and the nuclei in some primary spermatocytes were necrotic (Figure 8b). 

**Figure 9:**
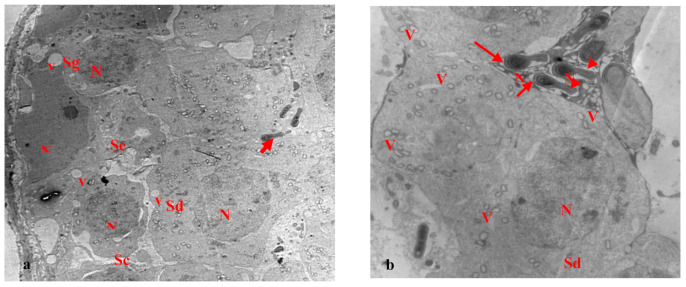
(**a**,**b**): Electron micrograph of a section of rats’ testis treated with EVOO and paracetamol for 15 days. (**a**) showing normal seminiferous tubule with normal spermatogonia (Sg), primary spermatocytes (Sc), spermatids (Sd) with normal nucleus (N). Sertoli cell (Se) appeared with flatted nucleus. Sperm appeared with elongated condensed nucleus and acrosomal cap (arrow) at the leading point of the head. Notice vacuoles (v) in some cells (×2000). (**b**) showing normal spermatids (Sd) and a normal nucleus. Normal sperm appeared with elongated condensed nucleus (N), acrosomal cap (arrow), and flagellum (head arrow). Notice vacuoles (v) in some cells (×2000).

The testis of rats treated with EVOO and paracetamol for 15 days showed normal seminiferous tubules with normal spermatogonia, primary spermatocytes, and spermatids. Sertoli cells appeared with flattened nuclei and the sperm had elongated condensed nuclei and acrosomal caps at the fronts of the heads (Figure 9a,b). Spermatids appeared with normal nuclei and vacuoles were observed in some cells (Figure 9b).

### 3.3. Hematological Results

**Figure 10:**
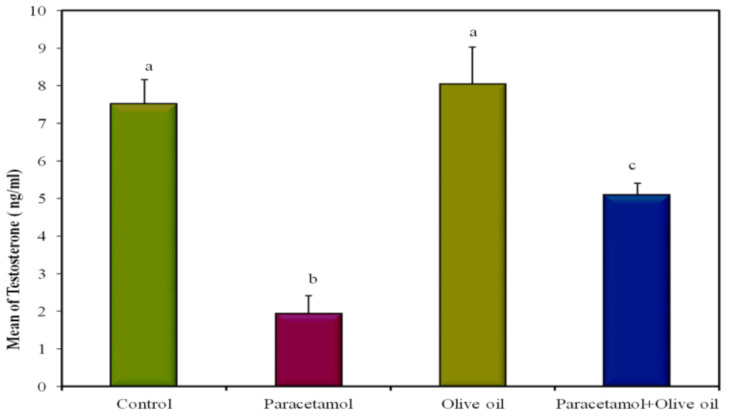
Effect of administration of paracetamol and/or EVOO on testosterone.

The serum testosterone (male sexual hormone) levels in the control group were compared to those in the paracetamol, EVOO, and paracetamol-with-EVOO groups (Table 2 and Figure 10) after 15 days of treatment. Table 1 shows statistically significant decreases in the testosterone levels in the paracetamol group when compared to the control and EVOO groups. Table 2 also shows that no statistically significant changes in testosterone levels were observed between the EVOO group and the control group after 15 days. However, a significant recovery and increase in testosterone levels was observed in the paracetamol-with-EVOO group compared to the paracetamol group. The results obtained indicate that EVOO protected the testis from toxicity induced by paracetamol and increased fertility in the male rats. 

Table 3 shows a significant decrease in the erythrocyte (RBC) count, hemoglobin content, and packed cell volume (PCV) in rats treated with paracetamol for 15 days compared to the control group. However, the erythrocyte count, hemoglobin contents, and PCVs of the EVOO group were similar to those of the control group. However, the data for the paracetamol-with-EVOO group showed an amelioration of these values compared to those of the paracetamol group. Table 3 shows no significant decrease in platelet counts between all the treatment groups and the control group. 

Furthermore, the total and differential leucocyte (WBC) counts in the male rats are shown in Table 4. The WBC counts significantly increased (*p* < 0.001) in the paracetamol group compared to the control and EVOO groups. However, the WBC count in the paracetamol-with-EVOO group clearly demonstrates amelioration compared to the paracetamol group.

Table 4 also shows that paracetamol administration resulted in a significant increase (*p*> 0.001) in stab cells compared to the control and other treatment groups, while other types of WBCs were slightly affected. Moreover, treatment with paracetamol caused nonsignificant changes in the neutrophils, monocytes, and eosinophils, which remained comparable to those in the control and other treatment groups, while it slightly decreased the lymphocyte count. Table 4 indicates that the total and differential WBC counts for the paracetamol-with-EVOO group are protected against the toxicity of paracetamol compared to those of the paracetamol group. 

**Figure 11:**
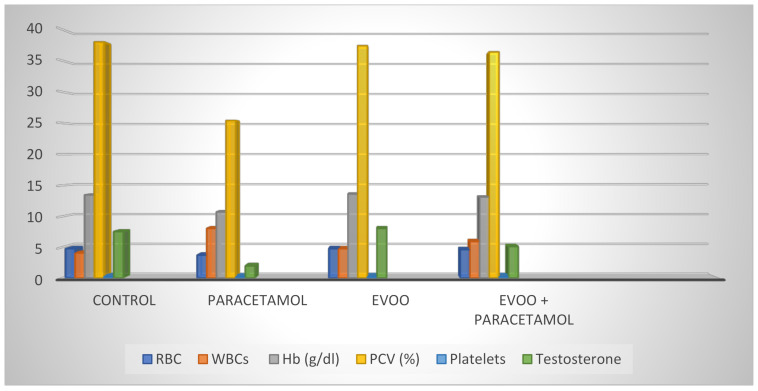
Protective effect of EVOO against paracetamol toxicity in blood and testis in male Wistar rats.

Table 5 and Figure 11 summarizes the protective effect of EVOO against paracetamol toxicity in blood cells, hemoglobin, and the testosterone parameters in male rats. All the histological, ultrastructural, and biochemical results obtained in the present study show that EVOO has the ability to protect the blood and testis against toxics when used in daily food. This amelioration is suggested to be due to the polyphenolic components in EVOO. The results also show that paracetamol decreased the PCV, Hb, and RBC counts, and significantly increased the WBC counts and stab forms. Moreover, paracetamol significantly decreases testosterone level. A significant increase in testosterone levels was observed in the testis of rats treated with EVOO and paracetamol. Light and electron microscopy showed disorganized sperms and seminiferous tubules in the testis of rats treated with paracetamol, whereas the sperms and seminiferous tubules appeared in normal shape in testis treated with EVOO and paracetamol.

## 4. Discussion 

Paracetamol is a widely used over-the-counter analgesic and antipyretic drug. Several studies have reported the toxicity of this drug with long-term use and overdoses in most of the body organs [2,3,4,5,6,7].

The histological and ultrastructural examination conducted in this study showed that treating rats with paracetamol for 15 days caused distortion and toxicity in the testis compared to those of the controls. Loss of the normal testicular structure was clearly observed, with markedly disorganized seminiferous tubules. Spermatogenic cysts were observed together with the separated and ruptured basement membranes of germinal epithelial cells and degenerated germ cells with pyknotic nuclei. Primary spermatocytes had pyknotic nuclei and rupture cell membranes. Moreover, wide separation was observed between the neighboring cells and the vacuoles for most cells. Most of the spermatogonia and primary spermatocytes showed chromatin condensation in their nuclei, and the nuclei in some primary spermatocytes were necrotic. Sertoli cells were observed with abnormal chromatin in their nuclei and basement membranes enclosing myoid cells. Some sperm had missing flagella with elongated condensed abnormal nuclei. 

A similar study found that treating experimental animals with paracetamol for 50 days induced many changes in the testicular structure, including altered seminiferous tubules and few degenerated tubules. Sertoli cells in tubules appeared fragmented, while the spermatids showed unusually well-developed rough endoplasmic reticular and Golgi complexes and had irregularly compacted chromatin. Late spermatids appeared to retain a larger volume of residual cytoplasm than those of the controls. The study suggests that these alterations may have been indirectly caused by dilated blood vessels and edema [27]. Similar results were obtained by El-Maddawy and El-Sayed [28] in rats treated with paracetamol. The study indicates that paracetamol induced oxidative damage and the impairment of the liver, kidney, and testicular functions, as well as hematotoxicity, which was confirmed by histopathological lesions, and increased serum aspartate aminotransferase, alanine aminotransferase, and alkaline phosphatase activities. A large single dose of paracetamol induced lipid peroxidation along with a significant decline in glutathione content and catalase activity in the liver, kidneys, and testicles. A paracetamol overdose resulted in decreased total serum protein, albumin, and globulin contents; increased bilirubin, urea, and creatinine contents; and induced hematotoxicity. Furthermore, paracetamol reduced sperm motility, sperm cell count, and the live sperm rate, and increased the sperm abnormality rate.

The hematological results obtained in the present study show significantly decreased testosterone levels in rats treated with paracetamol, indicating that the paracetamol may cause testicular toxicity and impaired fertility. Similar results were obtained by Luangpirom et al. [3] in male mice. This suggests that a high dose of paracetamol (0.05 g/100 g b.wt) induces testicular toxicity. A significant reduction in blood testosterone and impaired seminal quality were found in the paracetamol group. Another study investigated the toxic effects of a high dose of paracetamol on the reproductive system of male rabbits and showed similar results to those obtained in the present study. A significant decrease in blood testosterone and seminal quality impairment were observed in the group given high repeated therapeutic doses of paracetamol, which induced several changes and harmfully effected the histological structure of the seminiferous tubules. The study suggests that paracetamol can potentially cause reproductive toxicity and should be used cautiously, especially when high prolonged doses are indicated [29].

Moreover, the current study shows that paracetamol significantly decreased the erythrocyte (RBC) count, hemoglobin (Hb) content, and packed cell volume (PCV). This decrease in RBCs and Hb reduces the oxygen-carrying capacity of the RBCs and decreases the amount of oxygen reaching the cells, potentially causing anemia and impairing body functions. Similar reports show that treating rats with paracetamol decreased the PCV and RBC counts compared to the controls [4,30]. Moreover, the WBC and stab cell counts significantly increased in the paracetamol group compared to the control and other treatment groups. The present hematological results suggest that overdoses and long-term treatment with paracetamol may stimulate the immune system to protect the body from infections. The current results are consistent with a study published in 2015 [30], which investigated the effect of paracetamol on the hematological parameters in rats. The rats were given overdoses (300mg) of paracetamol for two days, and the results showed significantly increased WBC counts in the treatment groups compared to the control group.

A polyphenol in EVOO is considered to exhibit high antioxidant activity [31,32]. Thus, the present study investigated the protective effect of EVOO against paracetamol-induced hematotoxicity and testicular toxicity. The results obtained show amelioration in the paracetamol-with-EVOO group compared with the paracetamol group. The histological and ultrastructural examination show that most of the seminiferous tubules contained normal spermatogonia, primary spermatocytes, and spermatids. Sertoli cells had flattened nuclei and the sperm showed elongated condensed nuclei and acrosomal caps at the fronts of the heads. Spermatids appeared to have normal nuclei, and vacuoles were observed in some cells. The EVOO group showed improvements in most of the seminiferous tubules and less prominent histopathological alterations compared to the paracetamol group. This amelioration may be due to a polyphenolic component in EVOO and a potential indirect reduction of oxidative stress via gene expression modulation and enzyme activity, which enhances enzymatic antioxidant defenses [16]. A similar report [29] indicated that olive oil has a protective effect against oxidative damage induced by 2,4-Dichlorophenoxyacetic acid, which is mainly related to the antioxidant potential of its hydrophilic fraction. Furthermore, olive oil protected the reproductive organs from injury related to cadmium toxicity. It plays a significant role in ameliorating testicular damage by reducing oxidative stress [17]. However, EVOO may efficiently prevent the initiation of carcinogenesis in the body because concentrations effective against oxidative DNA damage are easily achieved with a normal intake of olive oil [33]. Moreover, many studies present evidence of the benefits of olive oil in relation to cardiovascular disease, reduction of the low-density lipoprotein cholesterol, and important biological and clinical effects related to the intake of olive-oil-rich diets on lipoprotein metabolism, oxidative damage, inflammation, endothelial dysfunction, blood pressure, thrombosis, and carbohydrate metabolism. VOO may play a protective role against cardiovascular disease and atherogenicity. Previous studies have shown the ability of phenol extracts in VOO to positively modulate the LDLR protein levels on the hepatocyte. [20,22,23,34]. Moreover, several studies clarify the mechanism behind the protective effects of EVOO phenols on the cardiovascular system, which suggests a role played, not only by hydroxytyrosol, but also by the entire pool of secoiridoid molecules. In addition, the molecular mechanisms of these EVOO phenol extracts explain the hypocholesterolemic activity observed so far in many studies [21,22,23]. Recent study results show that OO reinforced the antioxidant profile of the brain, blood, small intestine, and muscles, and it induced oxidative stress in the liver, spleen, pancreas, and heart. Moreover, the study reports that OO, although considered a nutritional source rich in antioxidants, exerts tissue-specific action when administered in vivo [35].

A recent study demonstrated a good correlation between the chemical characterization of EVOO extracts, a group of polyphenols (secoiridoids, lignans, and flavonoids), and their biological functions in terms of anti-inflammatory activity using cultured in vitro tissue. The specific OO polyphenol combination could be crucial for inducing the biological effects and, perhaps, reducing the risk of chronic inflammation that supports the development and the ever-increasing incidence of noncommunicable diseases nowadays [36].

From the present results, the author suggests more studies directed towards the identification of the EVOO phenolic components that affect testicular protection, and their role in male reproduction/fertility, with measurement of the nuclei and cell areas, and the capsid and flagella length in the sperms.

## 5. Conclusions

The results obtained in the current study show that paracetamol overdose can destroy the cells in the testis and affect the blood cells and other blood components. These effects may be caused by paracetamol toxicity.

The results also indicate that EVOO plays a significant role in improving and protecting the testis and blood against toxicity induced by a paracetamol overdose. This protective effect may be due to its high oleic acid content and the antioxidant effect of its polyphenols. The polyphenolic components may also, directly or indirectly, ameliorate oxidative stress and protect the testis and blood.

The present study shows that the use of EVOO in daily food has the ability to protect the organs against toxicity and that EVOO is one of the best oils for healthy food and good health suitable for daily use for all ages. 

Further studies are required to assess the protective effects of EVOO against toxins in different body organs, and to describe the other important components in EVOO that protect body health.

## Figures and Tables

**Table 1:** Morphological parameters considered in the cell study.

Cell Part	Morphological Parameters
Nucleus	Shape, Chromatin, Number of nucleoli
Cytoplasm	Morphology of organelles, Vacuoles, Lipid droplets
Plasma membrane	Shape, Basement membrane
Flagella	Shape

**Table 2:** Effect of administration of paracetamol and/or EVOO on testosterone concentration in male Wistar rats.

	Control	Paracetamol	EVOO	Paracetamol + EVOO	*p*
Testosterone (ng/mL)	7.52 ^a^ ± 0.64	1.94 ^b^ ± 0.47	8.04 ^a^ ± 0.98	5.10 ^c^ ± 0.30	<0.001 *

Data represented as mean ± SE. Different superscripts are significant. *p*: *p* value for F test (ANOVA) and significant between groups using post hoc test (LSD) *: Statistically significant at *p* ≤ 0.05 concentration in male rats. The values are expressed as means ± S.E (*n* = 5), significant difference at *p* < 0.05.

**Table 3:** Effect of administration of paracetamol and/or EVOO on hematological parameters in male Wistar rats.

	Control	Paracetamol	EVOO	Paracetamol + EVOO	*p*
RBC (10^6^/mL)	4.75 ^a^ ± 0.13	3.69 ^b^ ± 0.20	4.75 ^a^ ± 0.24	4.59 ^a^ ± 0.24	0.006 *
Hb (g/dl)	13.46 ^a^ ± 0.42	10.70 ^b^ ± 0.80	13.62 ^a^ ± 0.76	13.14 ^a^ ± 0.89	0.043 *
PCV (%)	38.00 ^a^ ± 1.64	25.40 ^b^ ± 2.06	37.40 ^a^ ± 2.29	36.40 ^a^ ± 2.25	0.002 *
Platelets (10^3^/mL)	262.60 ^a^ ± 16.23	213.20 ^b^ ± 17.78	258.20 ^a^ ± 15.23	259.80 ^a^ ± 16.04	0.103 *

Data represented as mean ± SE. Different superscripts are significant *p*: *p* value for F test (ANOVA) and significant between groups using post hoc test (LSD) *: Statistically significant at *p* ≤ 0.05.

**Table 4:** Effect of administration of paracetamol and/or EVOO on WBC total and differential counts in male Wistar rats.

	Control	Paracetamol	EVOO	Paracetamol + EVOO	*p*
WBCs (10^3^/mm^3^)	4040.0 ^a^ ± 166.13	8040.0 ^b^ ± 370.94	4740.0 ^a^ ± 297.66	5920.0 ^c^ ± 278.21	<0.001 *
Differential WBC count % Neutrophils	59.00 ± 1.70	58.60 ± 2.11	56.60 ± 2.27	58.40 ± 2.16	0.851
Stab forms	2.80 ^a^ ± 0.37	6.20 ^b^ ± 0.86	3.0 ^a^ ± 0.32	2.60 ^a^ ± 0.51	0.001 *
Lymphocytes	33.40 ^ab^ ± 1.91	28.40 ^a^ ± 2.09	36.0 ^b^ ± 1.90	33.80 ^ab^ ± 2.13	0.092
Monocytes	3.60 ± 0.51	5.20 ± 0.58	3.40 ± 0.51	4.00 ± 0.84	0.214
Eosinophils	1.20 ± 0.37	1.60 ± 0.40	1.00 ± 0.32	1.20 ± 0.49	0.758
Basophils	0.0 ± 0.0	0.0 ± 0.0	0.0 ± 0.0	0.0 ± 0.0	-

Data represented as mean ± SE. Different superscripts are significant *p*: *p* value for F test (ANOVA) and significant between groups using post hoc test (LSD) *: Statistically significant at *p* ≤ 0.05.

**Table 5:** Protective effect of EVOO against paracetamol toxicity in blood and testis in male Wistar rats.

	EVOO + Paracetamol	EVOO	Paracetamol	Control
RBC (10^6^/mL)	4.59 ^a^ ± 0.24	4.75 ^a^ ± 0.24	3.69 ^b^ ± 0.20	4.75 ^a^ ± 0.13
WBCs (10^3^/mm^3^)	5920.0 ^c^ ± 278.21	4740.0 ^a^ ± 297.66	8040.0 ^b^ ± 370.94	4040.0 ^a^ ± 166.13
Hb (g/dL)	13.14 ^a^ ± 0.89	13.62 ^a^ ± 0.76	10.70 ^b^ ± 0.80	13.46 ^a^ ± 0.42
PCV (%)	36.40 ^a^ ± 2.25	37.40 ^a^ ± 2.29	25.40 ^b^ ± 2.06	38.00 ^a^ ± 1.64
Platelets (10^3^/mL)	259.80 ^a^ ± 16.04	258.20 ^a^ ± 15.23	213.20 ^b^ ± 17.78	262.60 ^a^ ± 16.23
Testosterone (ng/mL)	5.10 ^c^ ± 0.30	8.04 ^a^ ± 0.98	1.94 ^b^ ± 0.47	7.52 ^a^ ± 0.64

Data represented as mean ± SE. Different superscripts are significant. *p*: *p* value for F test (ANOVA) and significant between groups using post hoc test (LSD) *: Statistically significant at *p* ≤ 0.05 concentration in male rats. The values are expressed as means ± S.E (*n* = 5), significant difference at *p* < 0.05.

## Data Availability

Not applicable.
